# Performance Enhancement of Vanadium Redox Flow Battery by Treated Carbon Felt Electrodes of Polyacrylonitrile using Atmospheric Pressure Plasma

**DOI:** 10.3390/polym12061372

**Published:** 2020-06-18

**Authors:** Chien-Hong Lin, Yu-De Zhuang, Ding-Guey Tsai, Hwa-Jou Wei, Ting-Yu Liu

**Affiliations:** 1 Institute of Nuclear Energy Research, Longtan 30546, Taiwan; zhuang@iner.gov.tw (Y.-D.Z.); dgtsai@iner.gov.tw (D.-G.T.); hwajou@iner.gov.tw (H.-J.W.); 2 Department of Materials Engineering, Ming Chi University of Technology, New Taipei City 24301, Taiwan

**Keywords:** vanadium redox flow battery, carbon felt, atmospheric plasma, polyacrylonitrile

## Abstract

A high-performance carbon felt electrode for all-vanadium redox flow battery (VRFB) systems is prepared via low-temperature atmospheric pressure plasma treatment in air to improve the hydrophilicity and surface area of bare carbon felt of polyacrylonitrile and increase the contact potential between vanadium ions, so as to reduce the overpotential generated by the electrochemical reaction gap. Brunauer-Emmett-Teller (BET) surface area of the modified carbon felt is, significantly, five times higher than that of the pristine felt. The modified carbon felt exhibits higher energy efficiency (EE) and voltage efficiency (VE) in a single cell VRFB test at the constant current density of 160 mA cm^−2^, and also maintains good performance at low temperatures. Moreover, the cyclic voltammetry (CV) and electrochemical impedance spectroscopy (EIS) analysis results show that the resistance between electrolyte and carbon felt electrode decreased. As a result, owing to the increased reactivity of the vanadium ion on the treated carbon felt, the efficiency of the VRFB with the plasma-modified carbon felt is much higher and demonstrates better capacity under a 100-cycle constant current charge-discharge test.

## 1. Introduction

The vanadium redox flow battery (VRFB) is a proven technology that has a number of key and promising advantages, which give it much promise as the future of energy storage systems with a good charge–discharge property, its long lifecycle, as well as being nonflammable and easily scalable with grid-scale potential [1,2,3]. The VRFB system consists of an energy management system (EMS) to control the power in and out, a battery management system (BMS) of two electrolyte tanks with V^2+^/V^3+^ and VO^2+^/VO_2_^+^ redox species in sulfuric or other acidic solutions with both negative and positive electrodes, and at least two pumps, as well as a battery stack where the key battery reaction takes place. The electrolyte is pumped into the stack and separated by the ion exchange membrane and fills the reaction area [4,5,6]. The electrode in the battery is used to conduct the electrons, provide the charge transfer platform, and make good contact with the electrolyte. For the VRFB system, the ideal electrode should have some essential properties, such as having good chemical stability in strong acid and redox reactions, good hydrophilicity, and lower electrochemical resistance [7,8,9,10,11,12], in order to obtain a reliable product that has higher voltage efficiency, charge capacity, and a longer lifecycle.

The electrode of VRFB often uses carbon materials such as carbon or graphite felt, paper, and cloth, which have excellent electronic conductivity and strong acid resistance because of their material composition [13,14]. The physical flexibility of the carbon material electrode can be compressed in the narrow electrode flow space and the good electronic properties mentioned above contribute to the low IR-drop (the voltage drop due to energy losses in a resistor) of the battery and the successful running of the battery during long operation cycles. Despite the graphite-based carbon electrodes having a number of benefits, there are still some drawbacks, including a highly hydrophobic surface and poor reaction surface area. The hydrophobic surface leads to poor contact with the vanadium ions in the aqua phase electrolyte, and the low reaction surface area limits the electron transfer efficiency between the electrode and the reaction species in the electrolyte [9], which can lead to an obvious decrease in voltage efficiency (VE), energy efficiency (EE), and capacity of the VRFB in operational conditions. It is possible to improve the performance and efficiency of the VRFB by increasing the surface water affinity or the surface area. Surface modifications to make the carbon material surface hydrophilic can be achieved by wet (acid, alkali), dry (plasma), and radiation treatments (laser, radiations), without affecting the supporting structural properties. Various carbon electrode modification procedures have been documented in past literature, including oxidative methods to increase the surface oxygen functional groups, such as acidic treatment [8], heat treatment [7,11,12], and electrochemical active treatment [15], or surface decoration methods to improve the reaction surface area or spots, such as pasting Bi nanoparticles [16] or carbon nanotube immobilization [17]. There are also other special methods, such as carving out laser pinholes [18], water–gas reaction methods, and plasma treatment methods [19,20].

**Figure 1:**
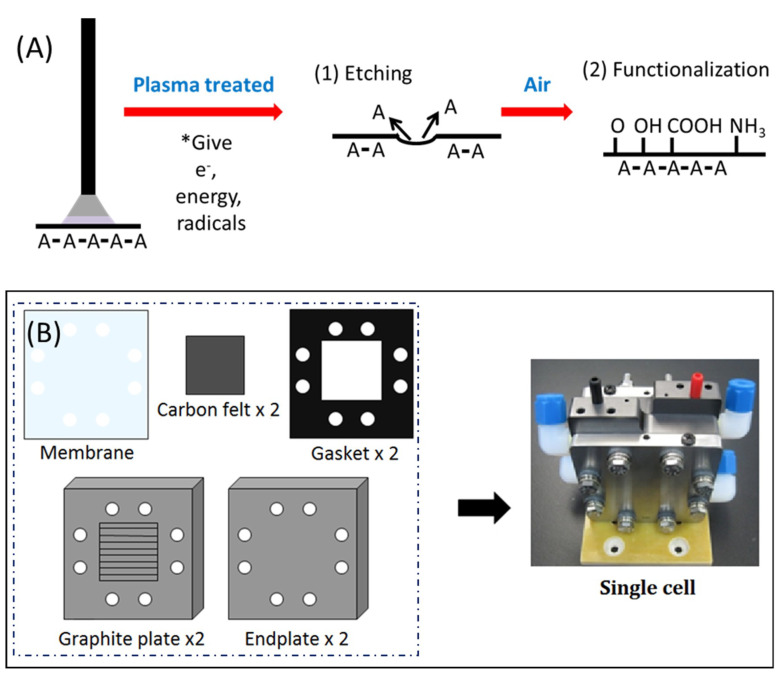
(**A**) Schematic diagram of how the plasma jet affects the surface of carbon felt. (**B**) Schematic illustration of single cell construction.

Atmosphere plasma treatment is an effective method for surface modification because it is solvent-free, dry, controllable, and easy to operate, with low or no waste [20]. For carbon materials, the main purpose of plasma treatment is the physical bombardment effect of the accelerated molecules, which effectively brings up the etching fragment and forms the carbon radicals on the carbon material surface [21,22,23], while still keeping the graphite backbone stable, as shown in Figure 1A. The etched carbon fiber surface significantly increases the surface area to improve the contact rate of the reaction species in the electrolyte of the batteries. The radicals formed by the plasma treatment on the carbon surface will change into oxygen-containing functional groups to increase the affinity of the aqua solution [20,24]. All of these benefits serve as a solution for improving the performance of the VRFB system, but an overetched electrode can lead to decreased conductivity and decreased performance. Thus, the modified conditions or methods are still being further investigated and developed.

In this work, the raw carbon felt was first treated by a nitrogen plasma jet under air and then the treated felt was exposed to air for a few minutes post-treatment. The radicals formed by the nitrogen plasma treatment on the surface of carbon felt will transfer into oxygen-containing functional groups after exposed in the air. This process is focused on solving the low electrochemical reactivity and the poor aqua affinity of the carbon felt electrode. Brunauer-Emmett-Teller (BET) examination showed that the treated felt had approximately 20 times higher BET surface area than the pristine felt, had become more hydrophilic, and had better reactivity within the vanadium electrolyte, shown using a water dropping test and electrochemical analysis methods such as cyclic voltammetry (CV), electro impedance spectrum (EIS), and single-cell VRFB test. Therefore, the atmosphere plasma jet treatment for preparing the modified carbon electrode is a very simple, well-established, and inexpensive technique, which can directly improve the performance of the VRFB cell without the need for other hardware changes.

## 2. Materials and Methods

### 2.1. Materials

Bare carbon felt material was purchased from CeTech co. Ltd., Taichung, Taiwan. Vanadium electrolyte for a single-cell test was purchased from Hong Jing environment, Pingtung, Taiwan. Vanadyl sulfate (VOSO4) for CV tests was purchased from Echo Chemical co. Ltd., Miaoli, Taiwan. The 99.999% pure nitrogen gas for the plasma treatment process was purified by the pressure swing adsorption (PSA) system (United Air System Co. Ltd., New Taipei City, Taiwan). All other chemicals, except for gases, were used as-received without further purification.

### 2.2. Preparation of Plasma-Treated Carbon Felt

Figure 1A depicts how the plasma jet affects the surface of the materials. The plasma-treated carbon felt electrode was prepared using the atmosphere plasma jet system with a rotating nozzle. Prior to modification, the plasma was generated using an atmosphere plasma generator (Plasmatreat GmbH, Steinhagen, Germany) at room temperature and the atmospheric environment. The purified nitrogen gas with an output at a pressure of 7 bars and a volume concentration of 99.999% as a further plasma gas source was produced from the PSA machine (United Air System Co. Ltd., New Taipei City, Taiwan) to prevent an unpredictable oxygen side effect. Bare carbon felt was placed under the fixed plasma jet nozzle at a distance of 10 mm and moved by a moving plate system at a constant speed of at least 2 mm sec^−1^.

### 2.3. Hydrophilicity Characterization

The surface hydrophilicity of the felt electrode was first tested by water drop. The data of contact angles were observed using a FTA-1000B contact angle goniometer (Ten Angstroms, Folio Instruments, Kitchener, ON, USA) at 25 °C.

### 2.4. BET Surface Area Analysis`

For the BET surface area test, a total of 10 g felt was cut into pieces to form the sample. ASAP2020 micromeritics^®^ (Micromeritics Instrument Corp., Norcross, GA, USA) was used as the measuring tool. The process of isothermal absorption line condition started from the degas process, followed by a measuring process set from relative pressure 0.1 to 1 under 77 K. The desorption process operated under reverse, at room temperature. The results were transferred to surface area data using the Brunauer-Emmett-Teller (BET) calculation model.

### 2.5. CV (Syclic Voltammetry) Analysis

For the cyclic voltammetry test, the felt was cut to 1 mm^2^ size and 6.5 mm thick as the sample, and Autolab^®^ (swiss) was used as the current provider and data collector. The three-electrode system was built for a positive test and negative test.

In the positive electrode test, 0.2M VOSO_4_ (purchased from Alfa Aesar, Echo Chemical Co. Ltd., Miaoli, Taiwan) solution was used as electrolyte, saturated calomel electrode (SCE) as the reference electrode (RE), and 1 mm thick platinum wire as the working electrode (WE) and counter electrode (CE). The starting voltage was autodetected by the tool, the voltage range was set from −0.5 V to 1.5 V, and a scan rate of 5 mV s^−1^ was chosen for the data collection.

In the negative electrode test, the electrolyte had a concentration of 0.17 M vanadium and a valance of 2.8–3, and a 2 M sulfuric acid diluted solution was made from the electrolysis reaction of the VRFB single-cell test. For the electrodes, the SCE was used as the RE, and 2 mm of thick glassy carbon electrode was used as the WE and CE to prevent the fast evolution of hydrogen within the analysis process. The starting voltage was also autodetected by the tool, the voltage range was set from −1.5 V to 0.5 V, and a scan rate of 5 mV s^−1^ was selected.

### 2.6. EIS Analysis

For electrical impedance spectrometer (EIS) test, the felt and the electrode features were the same as the previous CV tests, but used a different process. In the test, 0.2 M VOSO_4_ (purchased from Alfa) solution was used as the positive test electrolyte, with 0.17 M and 2.8–3 valance, and the vanadium and sulfuric acid mix solution made from the electrolysis reaction of the VRFB single cell test was used as the negative electrolyte. The SCE was used as the RE and a 2 mm thick glassy carbon rod used for the WE and CE. The starting voltage was automatically detected by the tool. EIS was performed, wherein an alternating current (AC) voltage of 10 mV in the frequency range of 10^5^–10^−2^ Hz was applied at the open circuit potential.

### 2.7. The Construction of the Single Cell of VRFB

Figure 1B depicts the construction of the single cell of VRFB. The single cell comprises two plastic plates with a 3 mm depth of flow field, two copper plates with 3 mm thickness, two embedded graphite plates of 6 mm thickness, and two stainless steel plates, which served as the endplates. There are also two gaskets that are 1 mm in thickness, two 25 cm^2^ treated or pristine carbon felt electrodes with a thickness of 6.5 mm, and an N212 Nafion^®^ membrane (purchased form Chemours, Taipei, Taiwan) for electrolyte separation.

### 2.8. VRFB Single-Cell Test

The VRFB single cell, as described above, was used for this test. In the charge-discharge tests, the solutions of 1.7 M V^3+^/VO^2+^ (with valance 3.5) and 5 M H_2_SO_4_ were used as the starting electrolyte in both the negative and positive electrodes. The carbon felt served as the electrode, and the graphite plates and copper plates served as the current collector. The active area of the electrode in the cell was 25 cm^2^. The volume of electrolyte in each half cell was 80 mL. The VRFB single cell was charged and discharged within the current density range of 80–200 mA cm^−2^ depending on the need. To protect the carbon felt and graphite plates from breaking under the high power, the VRFB cell was charged and discharged within the voltage limit of 1.6–0.7 V. The lifecycle test was conducted under a current density set to 120 mA cm^−2^ and the other described conditions, for at least 50 cycles.

## 3. Results and Discussion

### 3.1. The Plasma-Treated Process and Condition Decision

**Figure 2:**
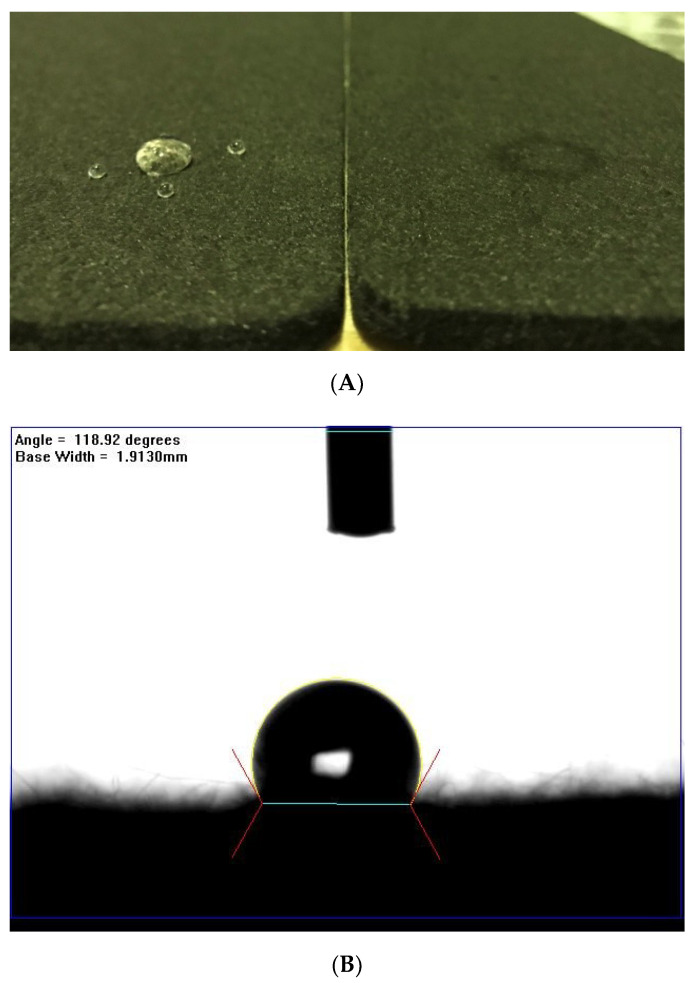

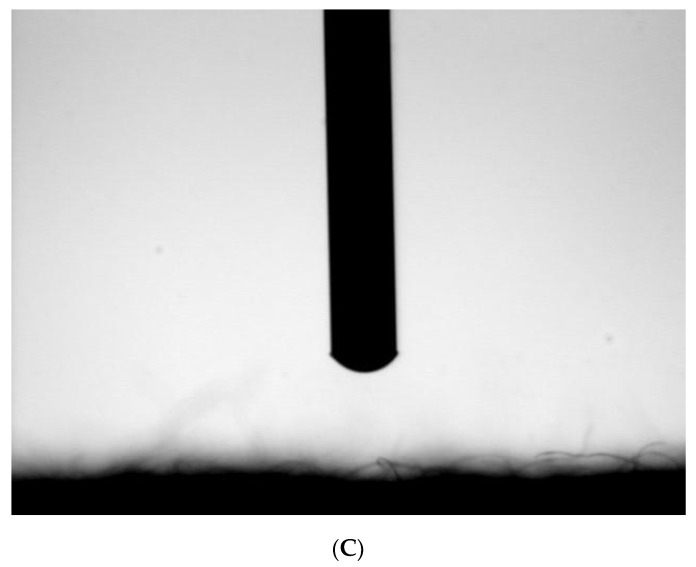
(**A**) The water dropping image of (left) the pristine felt and (right) the atmospheric plasma treated felt, the contact angle of (**B**) the pristine felt, and (**C**) the atmospheric plasma treated felt.

Carbon felt is an inert electrode that is difficult to modify. To break down the smooth carbon fiber surface or to introduce a functional group on it requires relatively high-energy reactions, such as the widely used plasma treatment methods, water-gas reactions, or electrochemical reactions between the carbon and chemicals. In this work, we used atmosphere pressure plasma as the treatment method because of its advantages of low temperature working conditions, being a fast treatment process, post-treatment free, and inexpensive. After treatment by the moving plasma jet at a velocity of 5 mm/s, keeping a 10 mm distance between the surface of the felt and the nozzle of the plasma jet, the surface hydrophilicity of the treated felt was determined by the water dropping method. Figure 2 indicates 118° ± 2° (Figure 2B) and ~0° (Figure 2C) of the contact angle on the pristine and treated felt surface, respectively, which may be attributed to the functional groups and defects formed by the free radical species reaction between the plasma species and carbon surface in the plasma jet. The result demonstrates how hydrophilic the treated felt had become. Moreover, it would be a great help to improve the pump loss of the VRFB stacks. In addition, the weight loss of the treated felt is less than 1%, which shows that the treated felt had broken down in some structures. The ash stacked in the plasma treatment process inside the chamber also proved that some destruction of the felt occurred. The thicknesses of the felt remained unchanged after plasma treatment, and it is thus directly ready to use.

**Figure 3:**
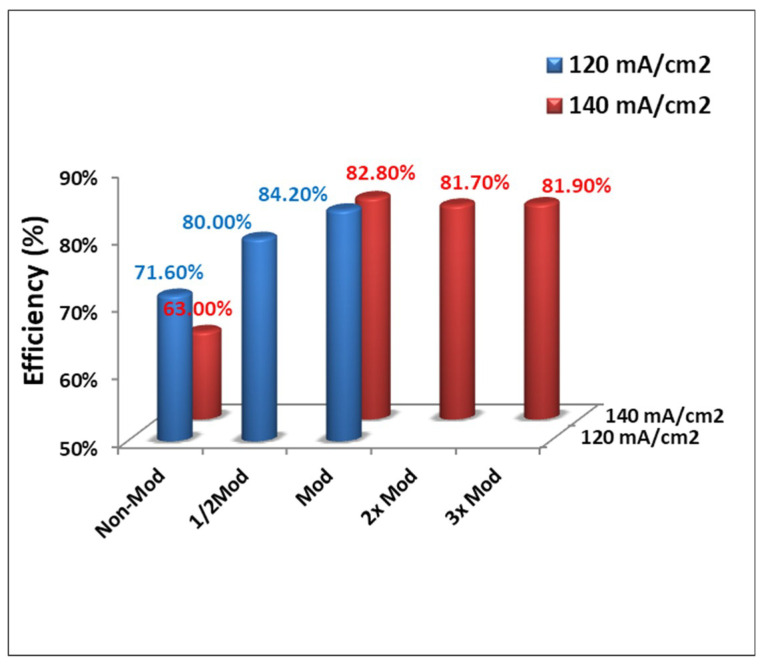
Efficiency (%) performance of the single cell equipped with the different parameters of atmospheric plasma treated carbon felt.

Furthermore, while the treatment may increase the hydrophilicity, it decreases the electronic conductivity of the felt. In other words, there is a tradeoff between electronic conductivity and electrochemical reactivity that should be carefully managed. In Figure 3, the single-cell was measured by carrying out 100 cycles of charge-discharge at a current density of 120 and 140 mA cm^−2^. The results of the average of EE suggest the modification process with the plasma jet at the relative velocity of 5 mm/s (EE_Avg._ = 84.2 ± 0.08%@120 mA cm^−2^ and EE_Avg._ = 82.8 ± 0.08%@140 mA cm^−2^) to be the best. Double speed plasma treatment (EE_Avg._ = 80.0 ± 0.05%@120 mA cm^−2^) and running the plasma treatment two times (EE_Avg._ = 81.7 ± 0.07%@140 mA cm^−2^) or three times (EE_Avg._ = 81.9 ± 0.08%@140 mA cm^−2^) did not deliver a better result.

### 3.2. The Surface Morphology Analysis

**Figure 4:**
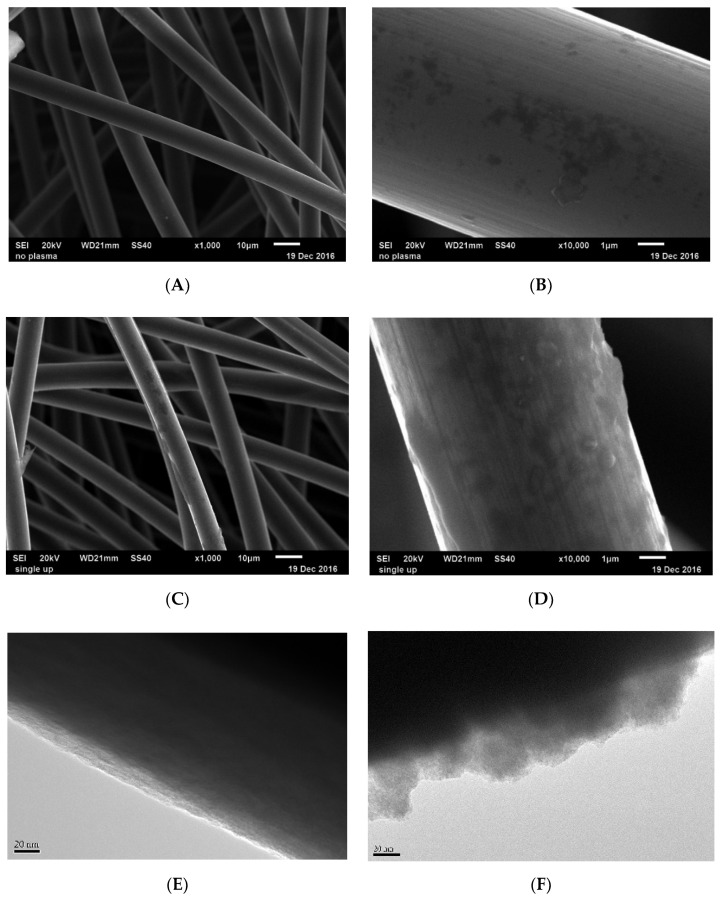
The SEM images of the pristine felt in (**A**) 1000 times zoom and (**B**) 10,000 times zoom; the atmospheric plasma treated felt in (**C**) 1000 times zoom and (**D**) 10,000 times zoom. The TEM images of (**E**) the pristine felt and (**F**) the atmospheric plasma treated felt.

To check the morphology changes in the pristine and treated felt surface, scanning electron microscope (SEM) and transmission electron microscopy (TEM) tools were used as the observation methods. Figure 4A–D depict the SEM images and Figure 4E,F depict the TEM images of the pristine felt and plasma-treated felt. The image (10,000 times zoom) of the plasma-treated felt (Figure 4D) shows that the defects on the carbon fiber surface were increased after the plasma treatment process. By contrast, the image of the pristine felt shows a smoother surface on the carbon fiber. Therefore, the roughness of the fiber surface increased after the plasma treatment, owing to the bombardment of accelerated heavy plasma species from the plasma jet.

### 3.3. BET Surface Area Analysis

**Figure 5:**
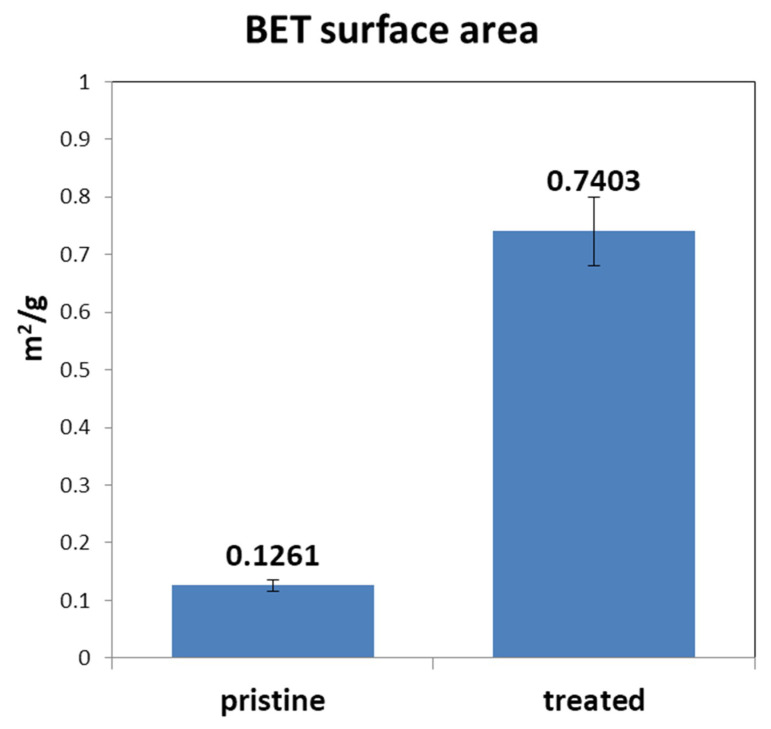
The diagram of the Brunauer–Emmett–Teller (BET) surface area results of the pristine and the atmospheric plasma treated felt.

The electrode reactive surface is an important issue as it affects the resistance of the electrochemical reaction, especially in a nonselective reaction system. In order to improve the electrochemical reaction efficiency between carbon felt electrode and vanadium ions in the electrolyte, we chose to increase the surface area of the felt. Figure 5 gives the comparisons of the pristine and the treated felt. The results of the tests, which were carried out under the same conditions, show that the BET surface area of the plasma-treated felt was approximately five times that of the pristine one. The measured surface area of the treated felt was 0.74 ± 0.06 m^2^ g^−1^ and the pristine one was only 0.13 ± 0.01 m^2^ g^−1^, although the surface area was very low for the BET model. The increasing surface area may also be attributed to the bombardment of the heavy plasma species in the plasma jet.

### 3.4. CV and EIS Analysis

**Figure 6:**
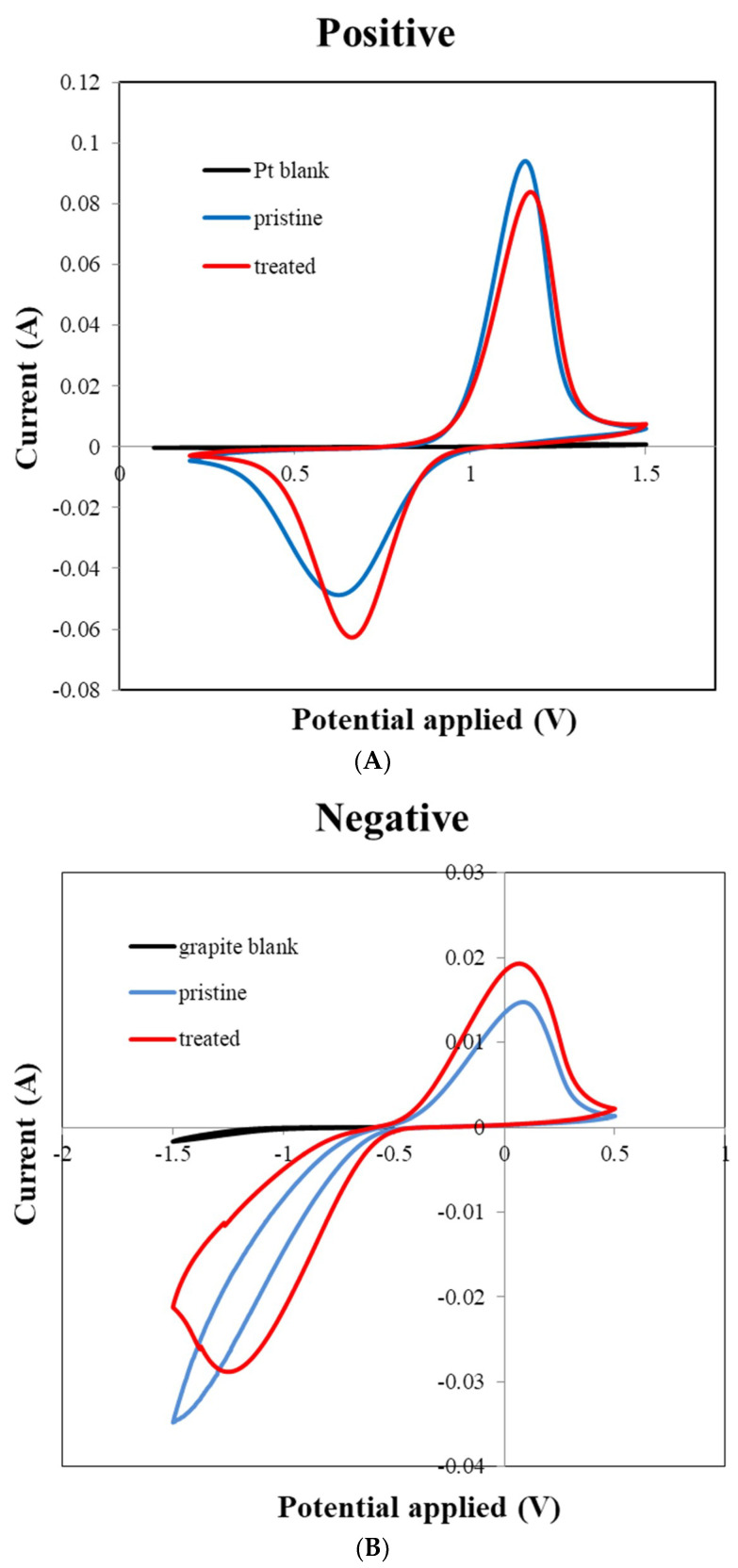
Cyclic voltammetry (CV) results of (**A**) positive electrodes (**B**) negative electrodes.

To observe the electrochemical property of the treated felt, both cyclic voltammetry (CV) and electro impedance spectrum (EIS) are good testing methods. The CV plot are the anodic peak current I_pa_, cathodic peak current I_pc_, anodic peak potential E_pa_, and cathodic peak potential E_pc_. Previous studies show that improved performance of the VRFB electrode is often indicated with an I_pc_/I_pa_ ratio close to 1 and a decreased ΔE value in CV examination, meaning that the reversibility of the redox reaction is improved [19]. Moreover, the Nyquist Plot by EIS analysis would have a smaller curve radius because of the decreased impedance of the felt or electrode after modification. In this case, Figure 6 shows the (A) positive and (B) negative electrode CV curves of the treated and pristine felts, which indicate a similar result to previous studies [15,16,17,18,19,20]. The positive electrode test result shows the decrease of the I_pc_/I_pa_ ratio from 1.93 to 1.34 and ΔE value from 0.532 V to 0.508 V, and the negative electrode shows the same trends, with the I_pc_/I_pa_ ratio increased from 0.424 to 0.669 and the ΔE value decreased from 1.582 V to 1.311 V. All of the results provide the evidence that the reversibility of the redox reaction to the felt electrode was improved after the plasma treatment process.

**Figure 7:**
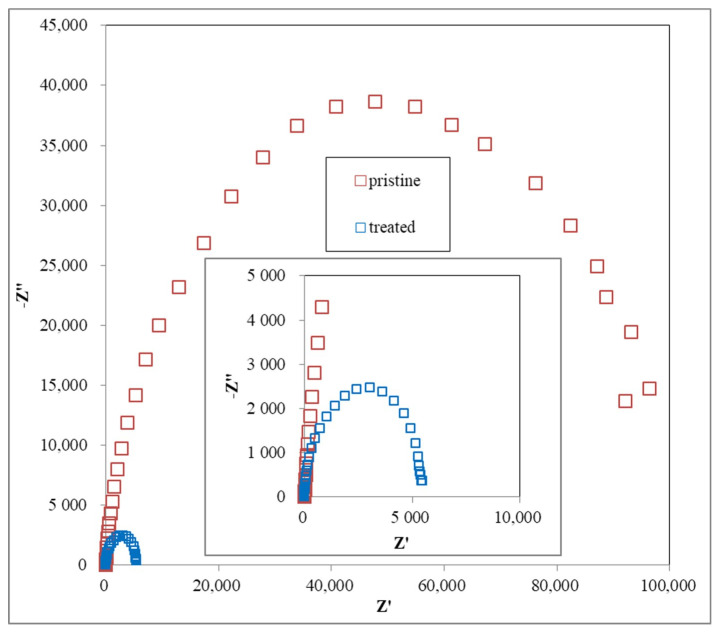
The Nyquist plots of the compared electrochemical impedance spectroscopy (EIS) results.

The Nyquist plots contain one semicircle in the high frequency range arising from charge transfer reactions at the electrolyte-electrode interface. The radius of the semicircle reflects the charge transfer resistance, with a smaller radius indicating a lower charge transfer resistance, which in turn indicates a faster electron transfer reaction [19]. EIS results (Figure 7) show that a smaller curve radius was found in the treated felt from the Nyquist plot compared with that in the pristine felt. It provides the evidence that the resistance of the felt used in the electrolyte system was decreased.

### 3.5. Charge-Discharge Curves

The charge-discharge curves of the second charge-discharge test cycle using the VRFB single cell often become the indication for cell performance comparison. Choosing the data of the second cycle of the test is owing to the unsteady electrolyte state in the first cycle, with a starting 3.5 valence vanadium electrolyte on both sides of the electrode. The test cell composed of Nafion 212 membrane was combined with the plasma-treated or the pristine carbon felt electrode to obtain comparable results.

**Figure 8:**
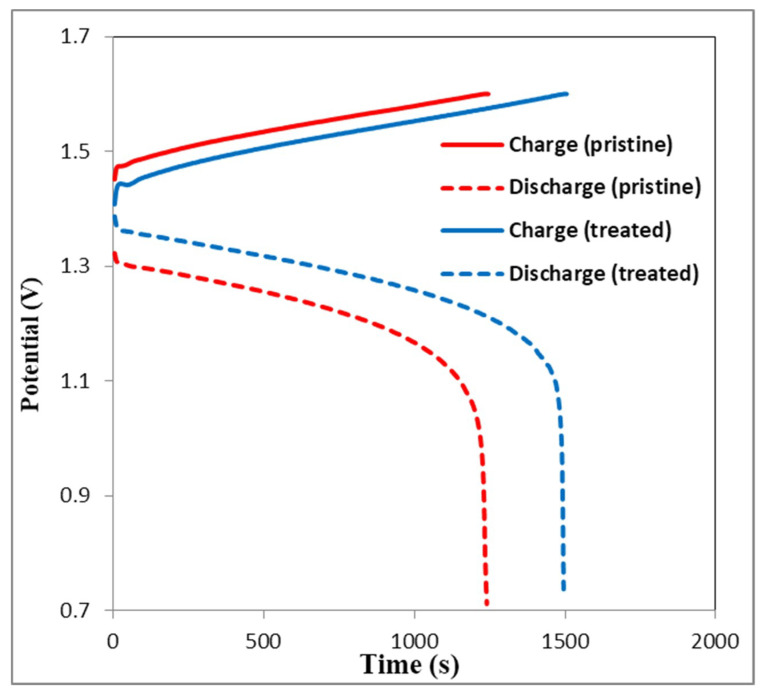
The comparison of second cycle charge-discharge curves of the carbon felt with and without atmospheric plasma treatments.

Figure 8 shows the charge-discharge curves of the second cycle of VRFB single cell with plasma-treated or the pristine carbon felt at 160 mA cm^−2^. It is obvious that the charge voltage of VRFB with plasma-treated felt is lower than that of the VRFB with pristine felt, while the discharge voltage of VRFB with plasma-treated felt is higher than that of the VRFB with pristine felt. While the discharge voltage trend is reversed, both results are attributed to the smaller IR drop of the treated felt. This result is likely caused by the lower area of resistance of the treated felt. This is because plasma treatment produces numerous oxygen-containing functional groups (such as –OH groups) on the surface of the carbon felts fibers, which are known to be electrochemically active sites for vanadium redox reaction. Furthermore, an increase of hydroxyl and carboxyl groups on the carbon felts fiber surface enhances its hydrophilicity, which makes it favorable for electrochemical reaction.

In addition, the data of the treated felt in a higher current density test provided a decreased capacity and EE owing to the stronger polarization effect, but it was still better than the pristine felt. Figure 8 shows the increased CE and VE results of the VRFB with the treated felt, which were 97.0% and 79.9% at the current density of 160 mA cm^−2^, respectively.

### 3.6. VRFB Single-Cell Performance

**Figure 9:**
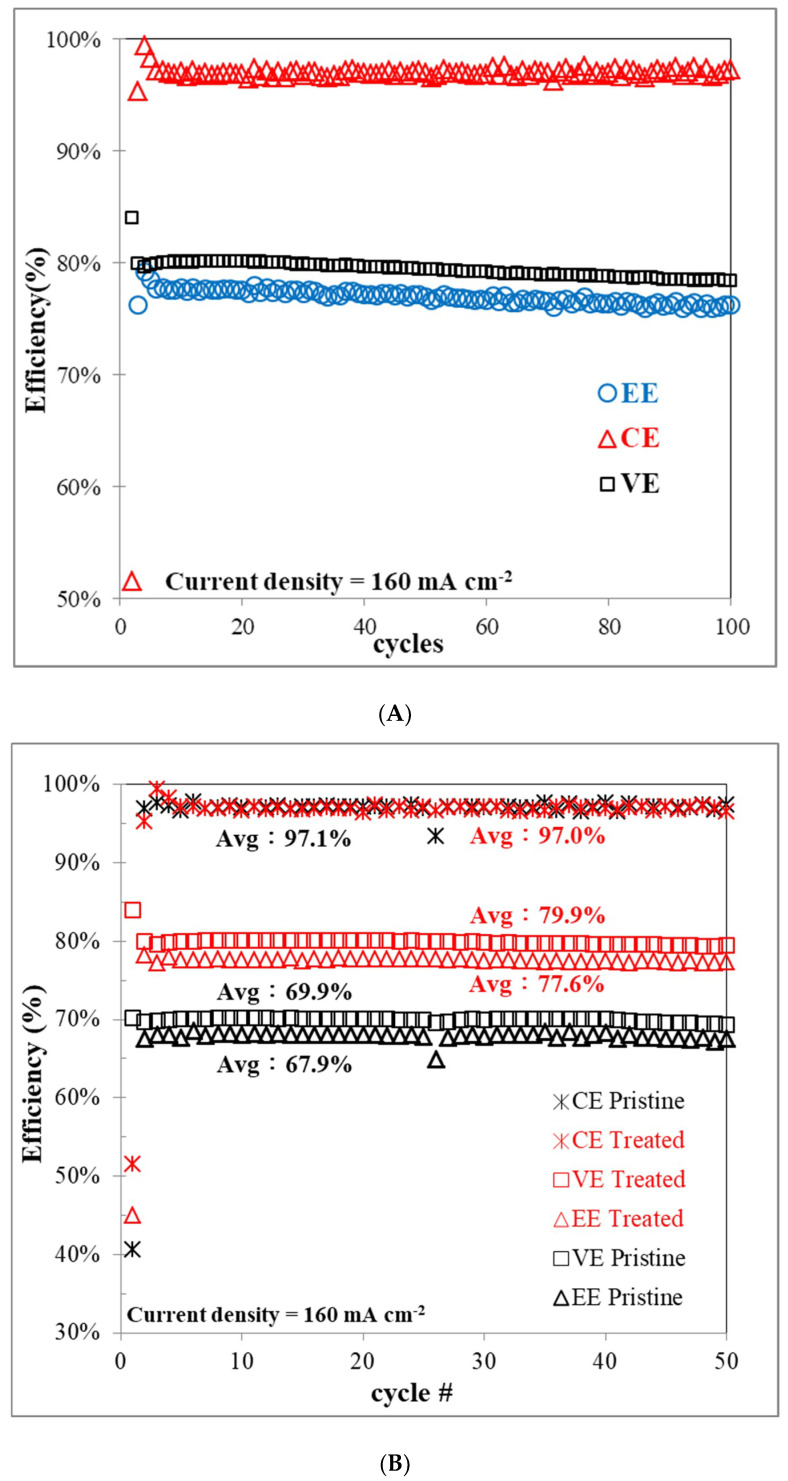

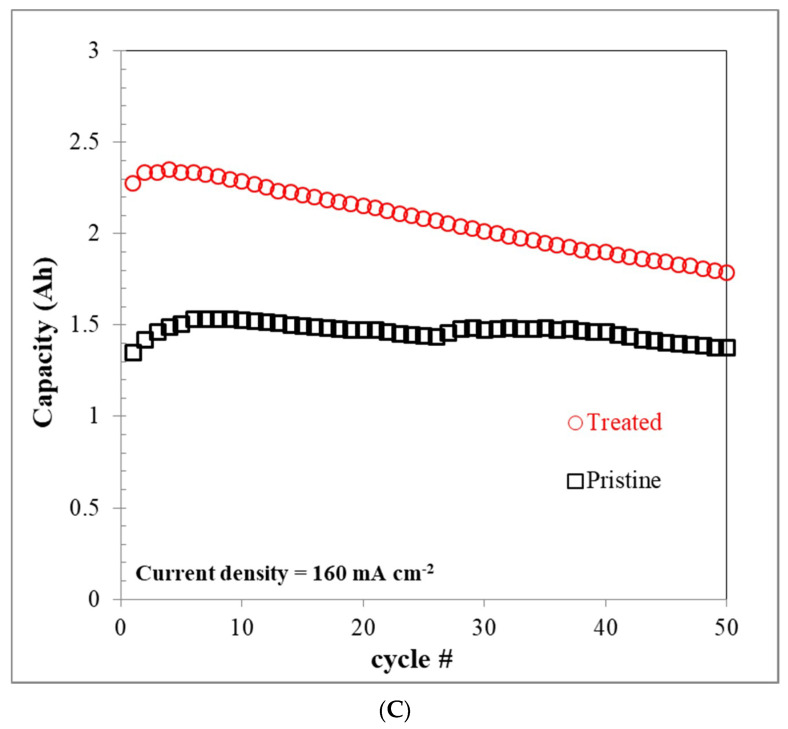
Diagrams of the performance of vanadium redox flow battery (VRFB). (**A**) One-hundred cycles of efficiency (%) performance of VRFB with the atmospheric plasma treated carbon felt. Fifty cycles of (**B**) efficiency (%) and (**C**) capacity (Ah) performance with and without atmospheric plasma treated carbon felts. EE, energy efficiency; VE, voltage efficiency; CE, coulombic efficiency.

A charge-discharge test was performed using a VRFB single cell to further demonstrate the effect of carbon felt on the electrochemical performance of the cell before and after plasma treatment. The in situ stability and performance test of the plasma-treated felt was carried out by a 100 cycle charge-discharge test using the VRFB single cell at the current density of 160 mA cm^−2^. The results shown in Figure 9A give the key performance values for the battery, which are the EE, CE, and VE. The curves of the above performance results remained smooth and stable for 100 cycles. The lack of decline in the performance indicated the high stability of the treated felt and also proved that the treated felt can remain stable in the strongly acidic and relatively high-oxidative vanadium electrolyte.

The performance of the EE value is the product of the VE and CE values. The increased VE value indicates the lower IR drop and thus the overpotential of the cell, and the increased CE value indicates the lower self-discharge that occurred in the test. The VE of the VRFB with the treated felt was higher than that of the VRFB with the pristine felt, at all current densities, which could be attributed to the reduced electrochemical resistance. The improved resistance of the felt electrode depends on two factors from the previous work the increased active surface area and the reduced area resistance [10]. Both of these aspects in the treated felt were improved, as shown by the surface area test, CV, and EIS analysis, thus demonstrating that the treated felt exhibited higher VE. The VRFB cell equipped with the treated felt has a greater VE than the pristine felt at all tested current densities, especially higher current densities, owing to the plasma treatment producing large amounts of oxygen-containing functional groups on the felt surface and promoting faster charge transfer, leading to improved electrode performance. In addition, the VE of the VRFB decreased with increasing charge-discharge current densities owing to the increase of ohmic resistance and the overpotential caused by the increase of current densities. The VE and EE are considerably higher for the VRFBs containing the plasma treated electrodes than the containing the pristine electrodes. Notably, these high efficiencies are maintained even at higher current densities.

In Figure 9B, the cell with the treated felt exhibited a higher performance than the pristine one in the same test conditions. It had the greater EE, which increased from 67.9% to 77.6%, and the capacity increased from 1.47 Ah to 2.08 Ah under the same constant current density and the other test conditions, which is more than 10% improvement. The higher average capacity of the 50 cycles test can be attributed to the improved hydrophilicity leading to the higher utilization rate of electrolyte and leading to the higher capacity of the VRFB under the same charge-discharge conditions.

The capacity curve (Figure 9C) of the treated felt showed a larger decreasing trend in the results, owing to the increasing migration of vanadium ions, hydrogen ions, and water in the electrolytes [9]. The imbalance of electrolytes increased faster than in the pristine felt by cycle number, because of the increased number of cycles completed on the treated felt. Therefore, the comprehensive performance increase in the VRFB single-cell test with the treated felt can be seen as important for future use in the scale-up stacks, as it will reduce costs because of requiring less electrolyte maintenance and having a higher electrolyte usage rate. Simple surface treatment of carbon felts using plasma treatment is thus promising for the assemblage of high-performance VRFBs, and we consider that this method is suitable for large-scale production of economical carbon felts electrodes.

## 4. Conclusions

In this study, the carbon felt electrode used for the VRFB cell was treated by an atmosphere plasma jet via a specific process and exhibited higher comprehensive cell performance than the pristine felt, thanks to its five times larger surface area and lower electrochemical resistance. The plasma treatment can also improve the hydrophilicity owing to the additional temperate water affinity functional group on the felt surface, which can reduce the contact angle to 0° and reduce the pumping loss when the VRFB system is operating. The single-cell test results with the treated felt from the charge-discharge cycling test shows that, even though the CE only had a small decrease, owing to the more than 20% improved capacity in the same test condition, the VE and the EE still increased significantly-up to 10% higher than the pristine felt under 160 mA cm^−2^ test conditions. The chemical stability of the treated felt tested by the 100 in situ charge-discharge cycle tests show the treated felt has high chemical stability in the vanadium electrolyte working environment. The results indicated that the hydrophilicity and electrochemical reaction of plasma-treated carbon felt electrodes can be greatly increased, which can improve the energy efficiency and capacity of carbon felt electrodes for VRFB. The facile and rapid surface treatment of carbon felt electrodes using atmospheric plasma would have potential to be applied in constructing the high-performance VRFB. Furthermore, we believe that the novel method is suitable for large-scale production of carbon felt electrodes, because the atmospheric plasma treatment industry is already well established.
